# Air Pollution in Space and Time: Birth Outcomes Are Complicated by Exposure Variations

**Published:** 2005-09

**Authors:** Bob Weinhold

The association between air pollution and adverse effects on human birth outcomes is drawing increasing attention worldwide. In one of the latest developments, two epidemiologists at the University of California, Los Angeles, have found that the typical use of air pollution data from fixed monitoring stations may be inadequate for accurately pinpointing the links between air pollution and birth outcomes **[*EHP* 113:1212–1221]**. They also corroborate earlier findings that timing of pollution exposures is significant and that studying different pollutant combinations substantially complicates analysis.

Using new data in a follow-up on previous work, the researchers discovered that carbon monoxide (CO) and particulate matter (PM_10_) had significant adverse effects—and at concentrations well below U.S. federal standards—on preterm and low-birth-weight (LBW) births for women living within one mile of an air pollution monitoring station. However, they measured substantially less or no effect for women living just two to four miles away. They also found the effects were most pronounced in association with exposure during early and late gestation, but less apparent for the full pregnancy.

The researchers used state and county databases documenting births to mothers living in the Los Angeles area from 1994 to 2000. To analyze LBW at full term, they studied a zip code cohort of 136,134 births, of which 2,778 were LBW. To analyze preterm births, they used 106,483 of the same births (minus births by cesarean section), of which 9,268 were preterm. Data from 18 government air pollution monitors documented CO, PM_10_, nitrogen dioxide, and ozone levels. There were two years of data for fine particulate matter (PM_2.5_), but the researchers found that wasn’t a long enough period to provide significant findings (although many other studies have found that PM_2.5_ is more of a health concern than PM_10_).

The researchers discovered that for the first trimester, the highest quartile of CO concentrations observed—never more than two-thirds of the U.S. Environmental Protection Agency (EPA) 8-hour standard—was associated with a 27% increase in risk of preterm birth for women living within one mile of a pollution monitoring station. Similar CO exposures and distances during the third trimester were associated with a 36% increase in LBW following a full-term pregnancy. Parallel effects were seen for PM_10_—which never exceeded two-thirds of the EPA 24-hour standard—early and late in pregnancy. This generally confirmed findings from an earlier study using data from 1989 to 1993. No significant relationships were seen for nitrogen dioxide or ozone.

The researchers were able to account for many potentially confounding factors, including maternal age, level of prenatal care, and infant’s sex. However, they had no data for other factors known to influence birth outcomes, such as maternal occupation, height, weight, weight gain during pregnancy, and smoking status. Folding in such data could affect the outcome of this study, the team acknowledges. The team’s finding that effect estimates diminished for women living farther than one mile from a station suggests that the air monitoring stations may not provide accurate measures of exposure for these women; development of ways to better capture spatial variability in pollutant concentrations is therefore an important goal.

The researchers concluded that improved air pollutant data reflecting both geographic variation and the specific substances in the ambient air mixture will lead to much better understanding of air pollutants on birth effects. Also important are the use of finer breakdowns of the pregnancy period and more detailed background information on the parents and child.

## Figures and Tables

**Figure:**
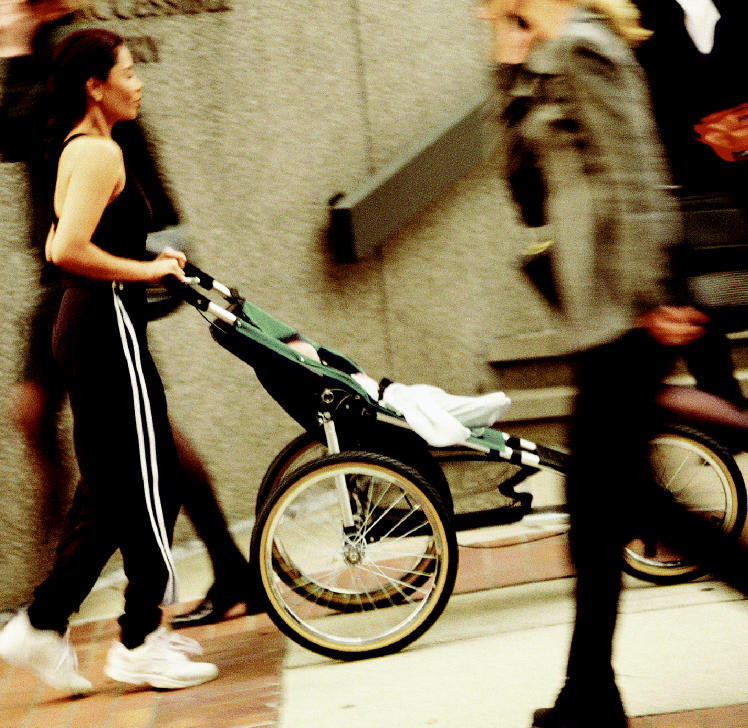
Born of necessity. A study of Los Angeles mothers shows that more detailed exposure information is critical for accurately drawing links between air pollution and adverse birth outcomes.

